# Mini-Sternotomy *vs.* Right Anterior Mini-Thoracotomy for Surgical Aortic Valve Replacement - A Systematic Review and Meta-Analysis

**DOI:** 10.21470/1678-9741-2024-0211

**Published:** 2025-03-11

**Authors:** Dimitrios Starvridis, Arian Arjomandi Rad, Paola Keese Montanhesi, Hristo Kirov, Max Wacker, Panagiotis Tasoudis, Murat Mukharyamov, Ricardo E. Treml, Jens Wippermann, Torsten Doenst, Ibrahim Sultan, Michel Pompeu Sá, Tulio Caldonazo

**Affiliations:** 1 Department of Cardiothoracic Surgery, University Clinic Magdeburg, Magdeburg, Germany; 2 Medical Sciences Division, University of Oxford, Oxford, United Kingdom; 3 Hospital Israelita Albert Einstein, São Paulo, São Paulo, Brazil; 4 Department of Cardiothoracic Surgery, Jena University Hospital, Jena, Germany; 5 Division of Cardiothoracic Surgery, University of North Carolina, Chapel Hill, North Carolina, United States of America; 6 Department of Anesthesiology and Intensive Care Medicine, Friedrich Schiller University Jena, Jena, Germany; 7 Department of Cardiothoracic Surgery, University of Pittsburgh, Pittsburgh, Pennsylvania, United States of America; 8 UPMC Heart and Vascular Institute, University of Pittsburgh Medical Center, Pittsburgh, Pennsylvania, United States of America

**Keywords:** Aortic Valve, Sternotomy, Thoracotomy, Reoperation, Aortic Valve, Constriction, Length of Stay, Stroke

## Abstract

**Introduction:**

Minimally invasive techniques for aortic valve replacement have become increasingly popular. The most common minimally invasive approaches are mini-sternotomy and right anterior mini-thoracotomy. We aimed to review the literature and compare clinical outcomes for these two approaches.

**Methods:**

Three databases were assessed. The primary endpoint was perioperative mortality. The secondary endpoints were reoperation for bleeding, stroke, operation duration, intensive care unit length of stay, cardiopulmonary bypass time, cross-clamping time, hospital length of stay, paravalvular leak, renal complications, conversion to full sternotomy, permanent pacemaker implantation, and wound infection. Random effects models were performed.

**Results:**

Ten studies were included in the meta-analysis (30,524 patients). There was no difference in perioperative mortality between groups (odds ratio: 0.83; 95% confidence interval 0.57-1.21; P=0.33). In comparison with mini-sternotomy, right anterior mini-thoracotomy showed higher rates of reoperation for bleeding (odds ratio: 0.69; 95% confidence interval 0.50-0.97; P=0.03), lower rates of stroke (odds ratio: 1.27; 95% confidence interval 1.01-1.60; P=0.04), and longer operation duration (standard mean difference: -0.58; 95% confidence interval -1.01 to -0.14; P=0.01). Other secondary endpoints were not statistically significant.

**Conclusion:**

The results suggest that both techniques present similar perioperative mortality rates for aortic valve replacement. However, right anterior mini-thoracotomy is associated with higher rates of reoperation for bleeding, lower rates of stroke, and longer operation duration time.

## INTRODUCTION

**Figure:**
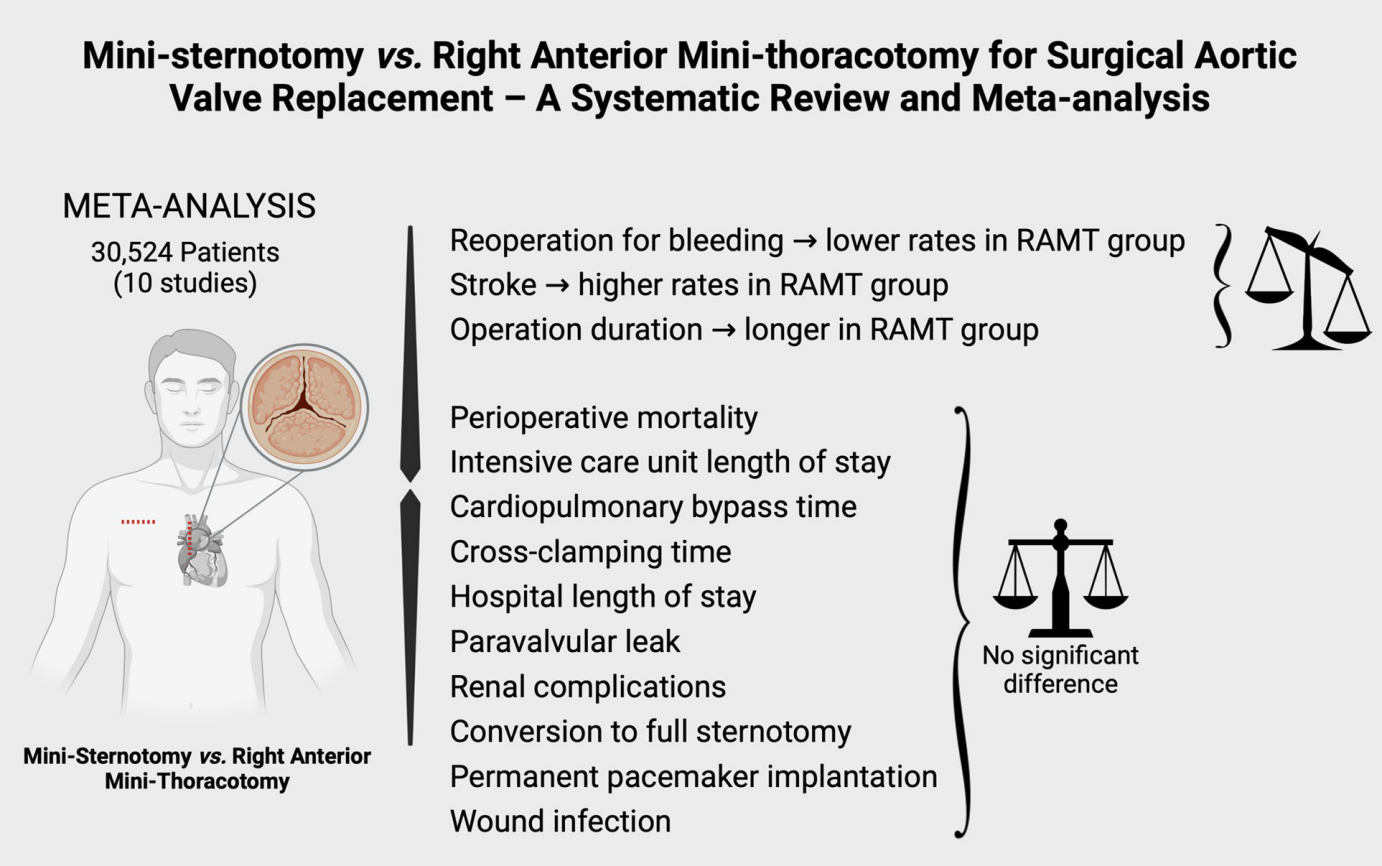

**Table:** 

Abbreviations, Acronyms & Symbols				
AVR	= Aortic valve replacement		M	= Multicenter
BMI	= Body mass index		MS	= Mini-sternotomy
CCTR	= Cochrane Central Register of Controlled Trials		NM	= Not multicenter
CI	= Confidence interval		NP	= Not prospective
CKD	= Chronic kidney disease		NR	= Not randomized
COPD	= Chronic obstructive pulmonary disease		OR	= Odds ratio
CPB	= Cardiopulmonary bypass		PAD	= Peripheral artery disease
EuroSCORE ICU	= European System for Cardiac Operative Risk Evaluation = Intensive care unit		PRISMA	= Preferred Reporting Items for Systematic Reviews and Meta-Analyses
LILACS	= Literatura Latino-Americana e do Caribe em Ciências da Saúde		RAMT SD	= Right anterior mini-thoracotomy = Standard deviation
LOS	= Length of stay		SMD	= Standard mean difference
LVEF	= Left ventricular ejection fraction			

Aortic valve replacement (AVR) have expanded dramatically over the years owing to advancements in treatment modalities and technologies^[1]^. Surgical options encompass conventional and minimally invasive approaches, both showing similar mortality rates^[2]^. Nevertheless, minimally invasive AVR has become increasingly popular due to its ability to avoid complete sternotomy. It offers several benefits, including reduced ventilation time, shorter intensive care unit (ICU) and hospital stays, lower blood loss and transfusion requirements, reduced atrial fibrillation rates, faster recovery, and better cosmesis^[3]^. Moreover, it is equally safe and efficient with reduced hospital costs^[4]^.

Mini-sternotomy (MS) and right anterior mini-thoracotomy (RAMT) are the most common minimally invasive approaches to AVR. These surgical techniques offer distinct advantages and technical challenges ranging from surgical exposure to postoperative recovery and outcomes. MS is performed through a 3- to 7-cm midline skin incision with upper partial sternotomy, and it is associated with less postoperative pain, less blood loss, and lower rates of wound infection and dehiscence^[2,3]^. Conversely, RAMT is performed through a 5- to 7-cm incision in the right second intercostal space without traumatizing the sternum. Comparative data showed lower postoperative pain and shorter ICU length of stay (LOS) with the use of thoracotomy^[5,6]^.

As one of the most performed procedures worldwide, AVR undergoes continuous refinement to improve surgical outcomes, minimize invasiveness, and optimize recovery. However, data comparing MS and RAMT are limited, and there are no randomized trials in the literature.

Salmasi et al.^[7]^ aggregated previously data on this topic showing significant differences regarding postoperative outcomes. Although its central aim was to compare directly these two techniques, this work has some intrinsic limitations that prevent it from providing a clear overview of this subject. For instance, a sensitivity analysis to identify outliers was not performed, and just < 10% of patients (n=2,926) from the current literature were included.

In this context, previous studies with populations basically from single-center registries have demonstrated the safety of RAMT in relation to perioperative mortality^[8]^, however a meta-analytical analysis involving all the major multinational registries on the subject based in the new standards of systematic reviews has not yet been performed. Ultimately, significant innovations marked the last 10 years of minimally invasive cardiac surgery and specially valve therapies. As the current guidelines do not mention any kind of procedure preference in different situations, choosing between the two approaches remains a surgeon’s decision and requires careful consideration.

In this context, we performed a systematic review of the topic and a meta-analysis of contemporary studies to compare major clinical outcomes between the two strategies.

## METHODS

Ethical approval of this analysis was not required as no human or animal subjects were involved. This review was registered with the National Institute for Health Research International Registry of Systematic Reviews (CRD42023451208).

### Search Strategy

We performed a comprehensive literature search to identify contemporary studies reporting shortand long-term outcomes between patients who underwent AVR with the two different techniques (MS or RAMT). Searches were run on March, 2023, in the following databases: Ovid MEDLINE®, Embase, and Google Scholar. The search strategy is available in Supplementary Table 1.

**Supplementary Table 1:** Complete search strategy.

Ovid MEDLINE®.
("aortic valve"[MeSH Terms] OR ("aortic"[All Fields] AND "valve"[All Fields]) OR "aortic valve"[All Fields]) AND ("replace"[All Fields] OR "replaceable"[All Fields] OR "replaced"[All Fields] OR "replaces"[All Fields] OR "replacing"[All Fields] OR "replacment"[All Fields] OR "replantation"[MeSH Terms] OR "replantation"[All Fields] OR "replacement"[All Fields] OR "replacements"[All Fields]) AND ("mini"[All Fields] AND ("sternotomy"[MeSH Terms] OR "sternotomy"[All Fields] OR "sternotomies"[All Fields])) AND ("mini"[All Fields] AND ("thoracotomy"[MeSH Terms] OR "thoracotomy"[All Fields] OR "thoracotomies"[All Fields]))
Translations
aortic valve: "aortic valve"[MeSH Terms] OR ("aortic"[All Fields] AND "valve"[All Fields]) OR "aortic valve"[All Fields] replacement: "replace"[All Fields] OR "replaceable"[All Fields] OR "replaced"[All Fields] OR "replaces"[All Fields] OR "replacing"[All Fields] OR "replacment"[All Fields] OR "replantation"[MeSH Terms] OR "replantation"[All Fields] OR "replacement"[All Fields] OR "replacements"[All Fields] sternotomy: "sternotomy"[MeSH Terms] OR "sternotomy"[All Fields] OR "sternotomies"[All Fields] thoracotomy: "thoracotomy"[MeSH Terms] OR "thoracotomy"[All Fields] OR "thoracotomies"[All Fields] Keywords used in Embase and Google Scholar (aortic valve replacement) AND (sternotomy) AND (thoracotomy)

### Study Selection and Data Extraction

The study selection followed the Preferred Reporting Items for Systematic Reviews and Meta-Analyses (PRISMA) strategy. After de-duplication (*i.e.*, exclusion of records with the same Digital Object Identifier, but with possibly minimal differences in title), the records were screened by two independent reviewers (DS and AAR). Any discrepancies and disagreements were resolved by a third author (TC). Titles and abstracts were reviewed against predefined inclusion and exclusion criteria. Studies were considered for inclusion if they were written in English and reported direct between patients who underwent AVR with the two different techniques (MS or RAMT). Animal studies, abstracts, case reports, commentaries, editorials, expert opinions, conference presentations, and studies which did not report the outcomes of interest were excluded. The full text was pulled for the selected studies for a second round of eligibility screening. References for articles selected were also reviewed for relevant studies not captured by the original search. Studies by the same author comprising the same population were critically analyzed to avoid population overlapping.

**Supplementary Fig. 1:**
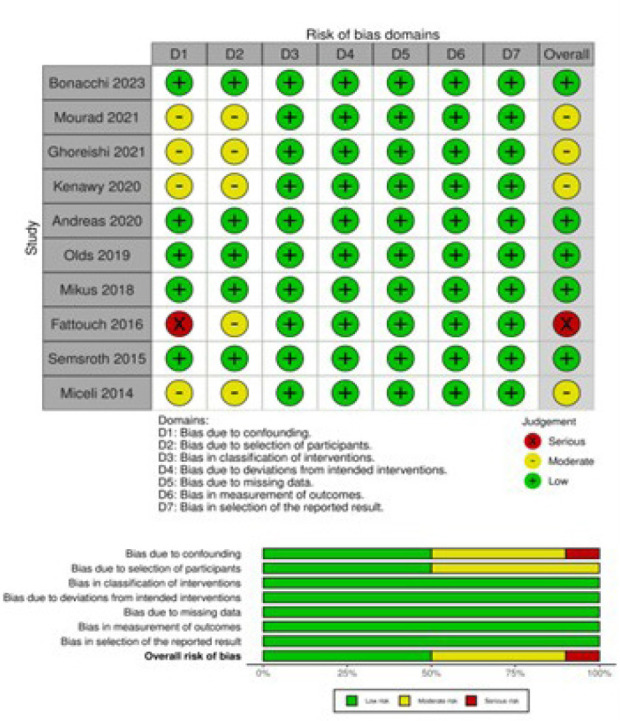
The Risk of Bias in Non-Randomized Studies of Interventions (or ROBINS-I) tool.

The Risk of Bias in Non-Randomized Studies of Interventions (or ROBINS-I) tool was systematically used to assess included studies for risk of bias^[9]^. The studies and their characteristics were classified into low, moderate, and serious risk of bias. Two independent reviewers (DS and AAR) assessed the risk of bias. When there was a disagreement, a third reviewer (TC) checked the data and made the final decision (Supplementary Figure 1).

Two reviewers (DS nd AAR) independently performed data extraction. Accuracy was verified by a third author (TC). The extracted variables included study characteristics (publication year, sample size, study design, country, study period, and presence or absence from population adjustment) as well as patient demographics (age, sex, body mass index [BMI], mean left ventricular ejection fraction [LVEF], European System for Cardiac Operative Risk Evaluation [EuroSCORE], hypertension, diabetes, chronic kidney disease, peripheral artery disease [PAD], chronic obstructive pulmonary disease [COPD], and nature of the aortic valve disease nature).

### Selected Endpoints

The primary endpoint was perioperative mortality, defined as 30-day or in-hospital mortality. The secondary endpoints were reoperation for bleeding, stroke, operation duration, ICU LOS, cardiopulmonary bypass (CPB) time, cross-clamping time, hospital LOS, paravalvular leak, renal complications, conversion to full sternotomy, permanent pacemaker implantation, and wound infection. Random effects models were performed.

### Statistical Analysis

Odds ratio (OR) with 95% confidence interval (CI) and *P*-values were calculated for each of the clinical outcomes. Standard mean difference (SMD) was calculated for the continuous variables. An OR > 1 indicated that the outcome was more frequently present in the MS group. A SMD > 0 corresponded to longer stay/time in the MS group. Forest plots were created to represent the clinical outcomes. Chi-squared and I2 tests were executed for the assessment of statistical heterogeneity^[10]^. By using a random effects model, the ORs were combined across the studies^[11]^. Inherent clinical heterogeneity between the studies was balanced via the implementation of a random effects model^[12,13]^. Funnel plots were constructed to assess publication bias. All analyses were completed through the “metafor” package of R Statistical Software (version 4.0.2), Foundation for Statistical Computing (Vienna, Austria).

## RESULTS

### Study Characteristics

**Fig. 1:**
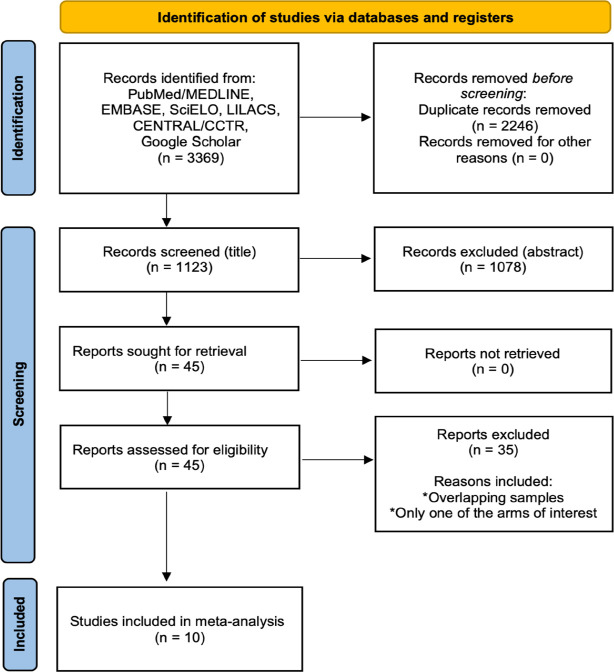
Preferred Reporting Items for Systematic Reviews and Meta-Analyses (or PRISMA) flow diagram. CCTR=Cochrane Central Register of Controlled Trials; LILACS=Literatura Latino-Americana e do Caribe em Ciências da Saúde.

A total of 1,123 studies were retrieved from the systematic search, of which 10 met the criteria for inclusion in the final analysis ^[8,14-22]^. Figure 1 shows the PRISMA flowchart for study selection.

Table 1 shows the details of the included studies. The overall patient number was 30,524, while MS patients were 20,932 and RAMT patients were 9,592. Included studies were published between 2014 and 2023, all the studies were non-randomized, observational, and with retrospective nature. Six studies included risk adjusted populations. Two studies were multinational databases.

**Table 1:** Summary of included studies.

Study	Patients (N) MS/RAMT	Study design	Country	Study period	Population comparability
Bonnachi et al. 2023^[15]^	1972 986/986	NR, NP, M	Italy	1999-2019	Propensity score matching
Mourad et al. 2021^[21]^	260 132/128	NR, NP, NM	Egypt	2014-2020	No adjustment
Ghoreishi et al. 2021^[17]^	23925 17306/6619	NR, NP, M	United States of America	2011-2017	Multivariable logistic regression
Kenawy et al. 2020 ^[18]^	232 126/106	NR, NP, NM	United Kingdom	2015-2019	No adjustment
Andreas et al. 2020 ^[14]^	1138 569/569	NR, NP, M	Multinational register	2013-2020	Propensity score matching
Olds et al. 2019 ^[8]^	387 267/120	NR, NP, NM	United States of America	2012-2015	Multivariable logistic regression
Mikus et al. 2018 ^[20]^	754 377/377	NR, NP, NM	Italy	2010-2017	Propensity score matching
Fattouch et al. 2016 ^[16]^	1130 854/276	NR, NP, M	Italy	2010-2014	No adjustment
Semsroth et al. 2015 ^[22]^	320 160/160	NR, NP, NM	Austria	2001-2012	Propensity score matching
Miceli et al. 2014 ^[19]^	406 155/251	NR, NP, NM	Italy	2005-2011	No adjustment

M=multicenter; MS=mini-sternotomy; NM=not multicenter; NP=not prospective; NR=not randomized; RAMT=right anterior mini-thoracotomy

### Patient Characteristics

Supplementary Table 2 summarizes the demographic data of the overall patient population. There was no relevant difference regarding age, sex, BMI, LVEF, EuroSCORE, presence of hypertension, diabetes, PAD, COPD, and combined aortic disease.

**Supplementary Table 2:** Patient demographics.

	Mini-sternotomy	Right Anterior Mini-thoracotomy
Age (years) Mean ± SD (total)	69.47 ± 11.72 (20785)	70.62 ± 11.46 (9739)
Female sex (%) (N/Total)	42.1% (8767/20785)	43.1% (4198/9739)
BMI (kg/m2) Mean ± SD (Total)	29.12 ± 5.89 (20510)	28.57 ± 5.87 (9221)
LVEF (%) Mean ± SD (Total)	59.12 + 9.81 (19645)	57.98 + 9.82 (9197)
EuroSCORE Mean ± SD (Total)	2.81 ± 3.03 (20367)	3.29 ± 3.0 (9345)
Hypertension (%) (N/Total)	74.4% (15484/20625)	73.8% (7074 / 9579)
Type II diabetes % (N/Total)	26.8% (5571/20785)	26.5% (2581/9739)
CKD % (N/Total)	5.5% (1078/19484)	10.5% (941/8924)
PAD % (N/Total)	10.1% (1916/18824)	13.4% (1111/8233)
COPD (%) (N/Total)	20.7% (4168/20092)	21.9% (1939/8827)
Aortic stenosis % (N/Total)	86.9% (17970/20659)	81.3% (7840/9633)
Aortic regurgitation % (N/Total)	64.6% (13346/20659)	57.3% (5520/9633)
Combined aortic disease % (N/Total)	24.7% (762/3073)	29.4% (762/2587)

BMI=body mass index; CKD=chronic kidney disease; COPD=chronic obstructive pulmonary disease; EuroSCORE=European System for Cardiac Operative Risk Evaluation; LVEF=left ventricular ejection fraction; PAD=peripheral artery disease; SD=standard deviation.

### Meta-Analysis

Central Image and Table 2 outline the detailed results of the meta-analysis.

**Table 2:** Outcomes summary.

Outcome	Number of studies	Number of patients	Effect estimate (95% CI; *P*-value)
Perioperative mortality	9	30,192	OR: 0.83; 95% CI 0.57-1.21; *P*=0.33
Reoperation for bleeding	9	29,396	OR: 0.69; 95% CI 0.50-0.97; *P*=0.03
Stroke	10	30,524	OR: 1.27; 95% CI 1.01-1.60; *P*=0.04
Operation duration	3	26,661	SMD: -0.58; 95% CI -1.01 to -0.14; *P*=0.01
Intensive care unit length of stay	9	30,118	SMD: 0.01; 95% CI -0.41 to 0.43; *P*=0.95
Cardiopulmonary bypass time	10	30,524	SMD: 0.32; 95% CI -0.72 to 1.36; *P*=0.54
Cross-clamping time	10	30,524	SMD: 0.32; 95% CI -0.72 to 1.36; *P*=0.53
Hospital length of stay	8	29,45	SMD: 0.00; 95% CI -0.34 to 0.34; *P*=0.11
Paravalvular leak	3	3,37	OR: 1.00; 95% CI 0.39-2.59; *P*=0.99
Renal complications	9	30,118	OR: 1.13; 95% CI 0.93-1.38; *P*=0.20
Conversion to full sternotomy	8	28,64	OR: 0.66; 95% CI 0.38-1.16; *P*=0.15
Permanent pacemaker implantation	6	5,052	OR: 0.83; 95% CI 0.48-1.44; *P*=0.50
Wound infection	8	29,731	OR: 1.05; 95% CI 0.54-2.05; *P*=0.88

CI=confidence interval; OR=odds ratio; SMD=standard mean difference

### Primary Endpoint

**Fig. 2:**
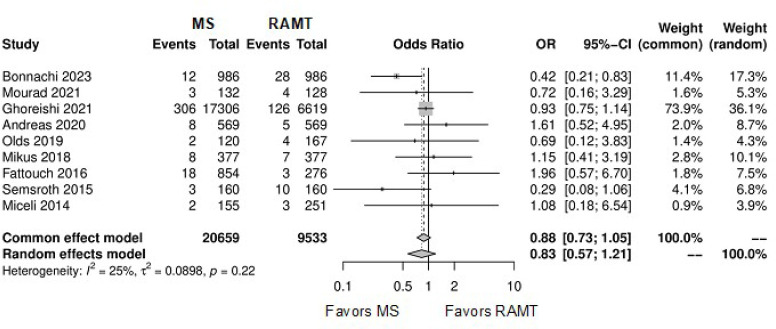
Forest plot for perioperative mortality. CI=confidence interval; MS=mini-sternotomy; OR=odds ratio; RAMT=right anterior mini-thoracotomy.

**Supplementary Fig. 2:**
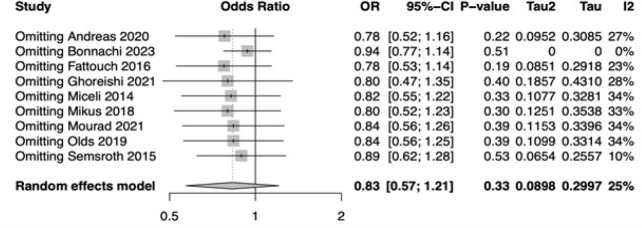
Leave-one-out analysis for perioperative mortality. CI=confidence interval; OR=odds ratio.

**Supplementary Fig. 3:**
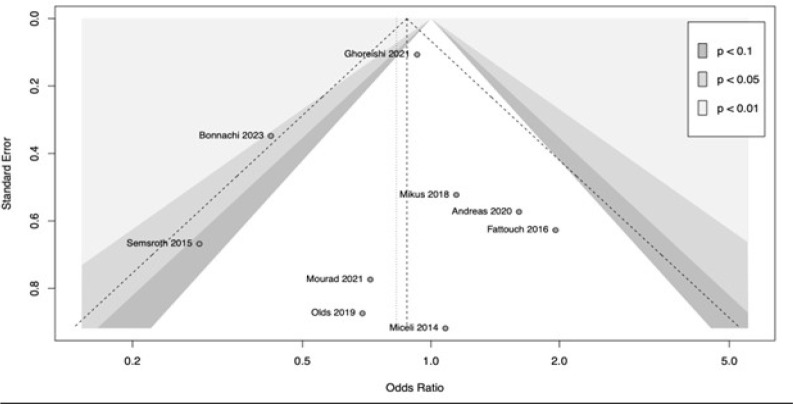
Funnel plot of the primary endpoint perioperative mortality.

Figure 2 shows the forest plot for perioperative mortality. There was not a statistical significance between the two approaches (OR: 0.83; 95% CI 0.57-1.21; *P*=0.33). Supplementary Figure 2 shows the leave-one-out analysis showing that most of the studies confirm the robustness of the analysis, with minimal variations of the CI. Supplementary Figure 3 provides the funnel plot for the publication bias assessment.

### Secondary Outcomes

**Fig. 3:**
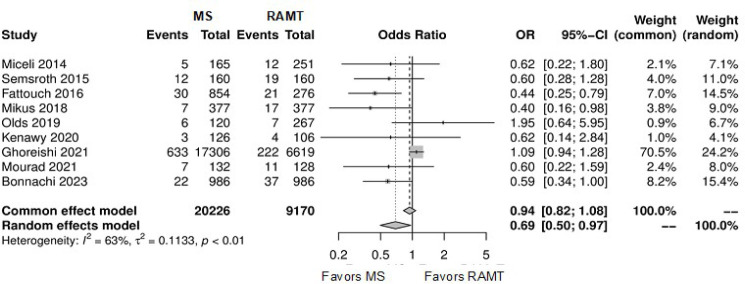
Forest plot for reoperation for bleeding. CI=confidence interval; MS=mini-sternotomy; OR=odds ratio; RAMT=right anterior mini-thoracotomy.

Figure 3 shows the forest plot for reoperation for bleeding. The RAMT group showed significantly higher rates of reoperation for bleeding in comparison with the MS group (OR: 0.69; 95% CI 0.50-0.97; *P*=0.03).

**Fig. 4:**
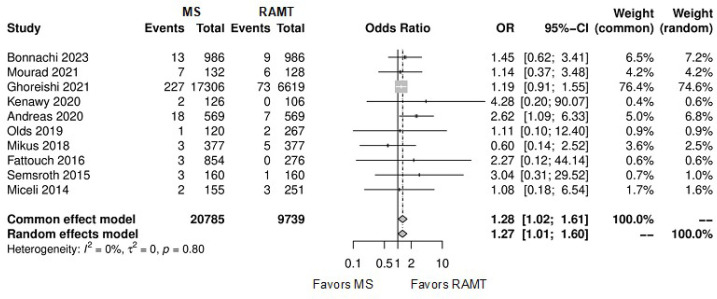
Forest plot for stroke. CI=confidence interval; MS=mini-sternotomy; OR=odds ratio; RAMT=right anterior mini-thoracotomy.

Figure 4 shows the forest plot for stroke. The RAMT group showed significantly lower rates of stroke in comparison with the MS group (OR: 1.27; 95% CI 1.01-1.60; *P*=0.04).

**Fig. 5:**
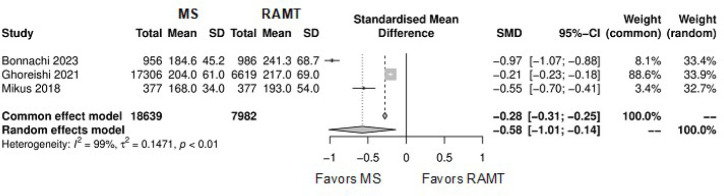
Forest plot for operation duration. CI=confidence interval; MS=mini-sternotomy; RAMT=right anterior mini-thoracotomy; SD=standard deviation; SMD=standard mean difference.

Figure 5 shows the forest plot for operation duration. The RAMT group showed significantly longer operation duration in comparison with the MS group (SMD: -0.58; 95% CI -1.01 to -0.14; *P*=0.01).

**Supplementary Fig. 4:**
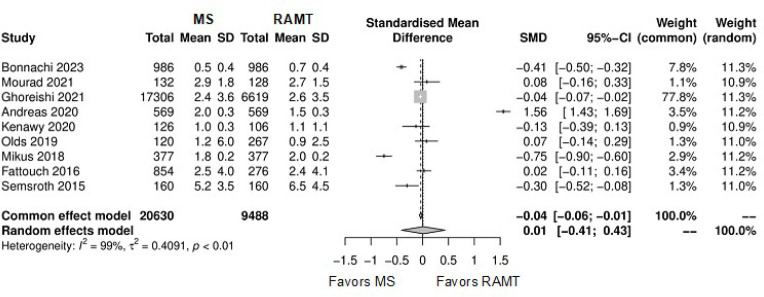
Funnel plot for intensive care unit length of stay. CI=confidence interval; MS=mini-sternotomy; RAMT=right anterior mini-thoracotomy; SD=standard deviation; SMD=standard mean difference.

**Supplementary Fig. 5:**
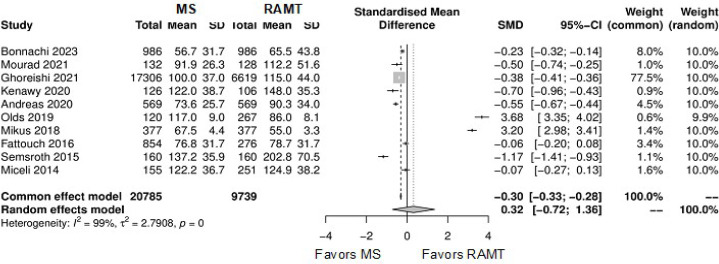
Forest plot for cardiopulmonary bypass time. CI=confidence interval; MS=mini-sternotomy; RAMT=right anterior mini-thoracotomy; SD=standard deviation; SMD=standard mean difference.

**Supplementary Fig. 6:**
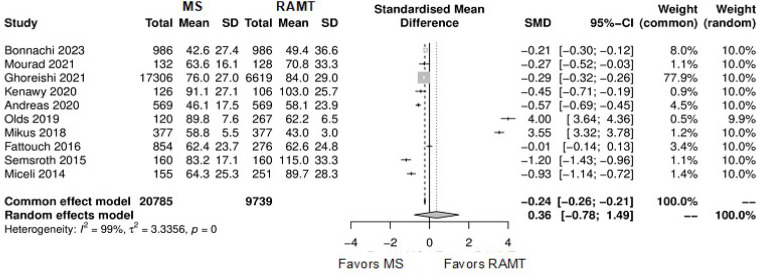
Forest plot for cross-clamping time. CI=confidence interval; MS=mini-sternotomy; RAMT=right anterior mini-thoracotomy; SD=standard deviation; SMD=standard mean difference.

**Supplementary Fig. 7:**
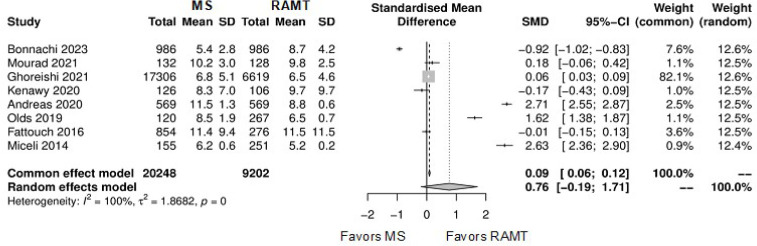
Forest plot for hospital length of stay. CI=confidence interval; MS=mini-sternotomy; RAMT=right anterior mini-thoracotomy; SD=standard deviation; SMD=standard mean difference.

**Supplementary Fig. 8:**
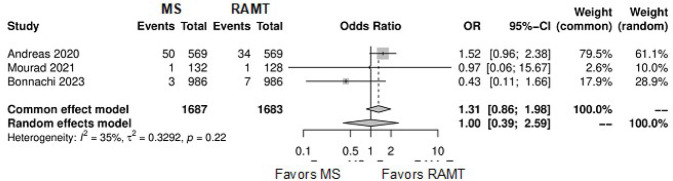
Forest plot for paravalvular leak. CI=confidence interval; MS=mini-sternotomy; OR=odds ratio; RAMT=right anterior mini-thoracotomy.

**Supplementary Fig. 9:**
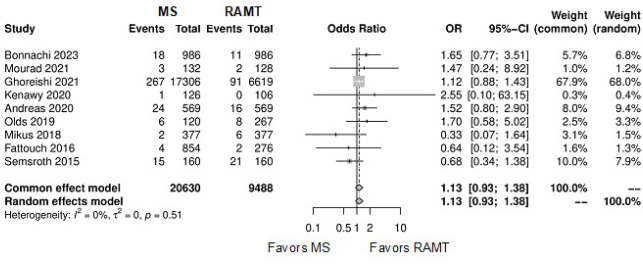
Forest plot for renal complications. CI=confidence interval; MS=mini-sternotomy; OR=odds ratio; RAMT=right anterior mini-thoracotomy.

**Supplementary Fig. 10:**
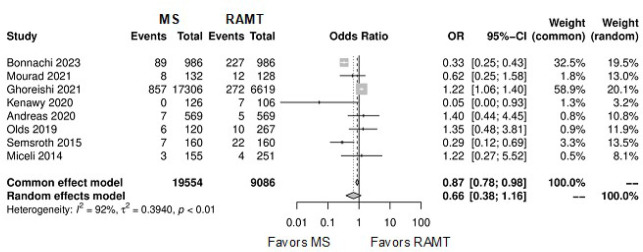
Forest plot for conversion to full sternotomy. CI=confidence interval; MS=mini-sternotomy; OR=odds ratio; RAMT=right anterior mini-thoracotomy.

**Supplementary Fig. 11:**
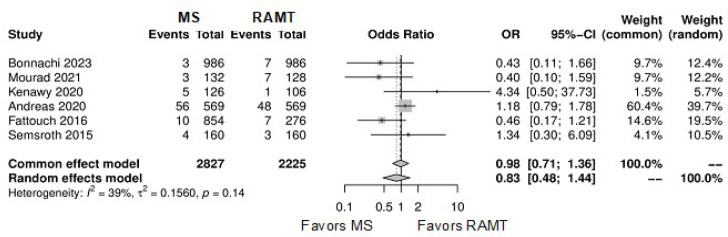
Forest plot for permanent pacemaker implantation. CI=confidence interval; MS=mini-sternotomy; OR=odds ratio; RAMT=right anterior mini-thoracotomy.

**Supplementary Fig. 12:**
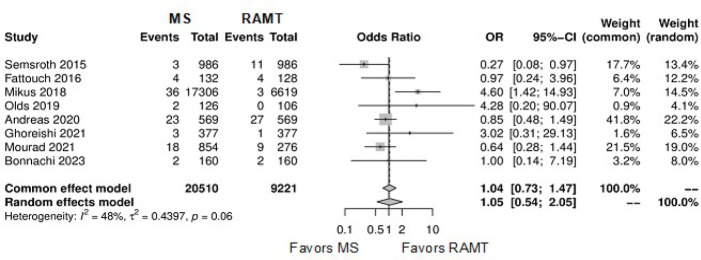
Forest plot for wound infection. CI=confidence interval; MS=mini-sternotomy; OR=odds ratio; RAMT=right anterior mini-thoracotomy.

Further comparison regarding ICU LOS, CPB time, cross-clamping time, hospital LOS, paravalvular leak, renal complications, conversion to full sternotomy, permanent pacemaker implantation, and wound infection were not statistically significant (Supplementary Figures 4-12). Information regarding the valve types “sutureless *vs.* stented” or “tissue *vs.* mechanic” were only mentioned in two of the studies^[14,15]^.

## DISCUSSION

The analysis suggests that both techniques present similar perioperative mortality rates for AVR. However, RAMT is associated with higher rates of reoperation for bleeding, lower rates of stroke and longer operation duration time. There was no difference regarding ICU LOS, CPB time, cross-clamping time, hospital LOS, paravalvular leak, renal complications, conversion to full sternotomy, permanent pacemaker implantation, and wound infection.

Minimally invasive alternatives for AVR have demonstrated comparable safety and efficacy to conventional median sternotomy^[6,8]^. Their ability to provide favorable clinical outcomes is associated with advancements in surgical techniques, instrumentation, and perioperative care^[6,8]^. This paradigm shift towards less invasive approaches has been driven by the pursuit of reduced morbidity, shorter hospital stays, expedited recovery, and greater patient satisfaction. Although this meta-analysis did not show any advantage in relation to any of the minimally invasive techniques when compared to each other, there is a relative reduction in postoperative pain associated with faster mobilization and recovery when comparing these techniques to traditional AVR with complete sternotomy^[23-25]^.

Previous comparisons between MS and RAMT provided valuable insights into their respective benefits and limitations^[8,14-22]^, but the best approach remains on debate. This systematic review and meta-analysis demonstrated equivalent perioperative mortality rates between the two groups, what emphasizes the competency of surgeons in performing both techniques and the overall safety of minimally invasive AVR.

Additionally, RAMT showed a higher rate of reoperation for bleeding when compared to MS. This finding suggests that MS may offer more effective hemostasis strategies due to better visualization, resulting in fewer postoperative bleeding complications. However, it does not decrease the safety of RAMT, as the bleeding events were not related to higher perioperative mortality in this analysis. The information is, however, relevant for special patient populations, for instance, Jehovah’s witnesses.

An important and curious fact about the work is the fact that MS was associated with more frequent stroke events. Although both techniques demand aortic cross-clamping for the surgery, the majority of RAMT procedures are performed using femoral cannulation, and it could be unexpectedly related with the reduction of cerebrovascular events^[26,27]^.

Furthermore, the longer operation times observed with RAMT may be attributed to the learning curve associated with this technique. As surgeons become more experienced with RAMT, operation times could potentially decrease, enhancing overall surgical efficiency^[28,29]^.

The choice between MS and RAMT is influenced by various factors, including surgeon experience and patient-specific considerations. The analysis revealed that most of the included studies had larger cohorts of MS patients, possibly indicating a preference for this technique among surgeons due to familiarity or perceived ease of execution. This raises questions about the impact of surgeon experience on the outcomes and preferences for a specific technique.

It's worth acknowledging that the lack of standardized reporting regarding valve types and replacement methods introduces heterogeneity into the analysis. The type of valve used and the method of replacement can also influence operation duration and, consequently, patient outcomes. As newer technology and instruments continue to develop, the creation of modern rapid deployment stented valves and refined instrumentation can contribute to enhanced patient safety and long-term outcomes for minimally invasive AVR. Finally, as transcatheter aortic valve replacement gains prominence, there is an increasing demand for surgical solutions that offer superior cosmetic results, shorter hospital stays, and long-term durability. The insights gained from this analysis contribute to the ongoing dialogue surrounding the selection of surgical approaches, particularly in the context of evolving patient expectations and advancing technology.

### Study Strength and Limitations

We analyzed 12 different outcomes besides mortality. Six out of ten studies presented data from risk-adjusted populations and most of them had a well-designed methodological approach. However, this work has the intrinsic limitations of observational series, including the risk of methodological heterogeneity of the included studies and residual confounders. In addition, treatment allocation bias is likely present in all observational series comparing two therapies with different invasiveness. Ghoreishi et al. provided a significant number of patients compared to the other studies^[17]^. Finally, information regarding whether the same surgeons performed both techniques or not was not mentioned in all of the studies. Besides that, there was not enough data in the studies regarding the type of prosthesis used, which can substantially influence long-term results, and regarding the complications resulting from peripheral cannulation. Precisely for this reason, this work has an important role in generating hypotheses and indicating possible clinical correlations between clinical events that can definitely support the design of new RCTs. Therefore, this meta-analysis based on observational studies has per se the limitation of not being able to produce relationships with a causal character.

## CONCLUSION

The analysis suggests that both techniques present similar perioperative mortality rates for AVR. However, RAMT is associated with higher rates of reoperation for bleeding, lower rates of stroke, and longer operation duration time. There was no difference regarding ICU LOS, CPB time, cross-clamping time, hospital LOS, paravalvular leak, renal complications, conversion to full-sternotomy, permanent pacemaker implantation, and wound infection.
